# The Dynamics of Somatic Exocytosis in Monoaminergic Neurons

**DOI:** 10.3389/fphys.2012.00414

**Published:** 2012-11-06

**Authors:** Bidyut Sarkar, Anand Kant Das, Senthil Arumugam, Sanjeev Kumar Kaushalya, Arkarup Bandyopadhyay, Jayaprakash Balaji, Sudipta Maiti

**Affiliations:** ^1^Department of Chemical Sciences, Tata Institute of Fundamental ResearchMumbai, India

**Keywords:** confocal microscopy, depolarization, multiphoton imaging, neurotransmission, serotonin, total internal reflection fluorescence microscopy, vesicle dynamics

## Abstract

Some monoaminergic neurons can release neurotransmitters by exocytosis from their cell bodies. The amount of monoamine released by somatic exocytosis can be comparable to that released by synaptic exocytosis, though neither the underlying mechanisms nor the functional significance of somatic exocytosis are well understood. A detailed examination of these characteristics may provide new routes for therapeutic intervention in mood disorders, substance addiction, and neurodegenerative diseases. The relatively large size of the cell body provides a unique opportunity to understand the mechanism of this mode of neuronal exocytosis in microscopic detail. Here we used three photon and total internal reflection fluorescence microscopy to focus on the dynamics of the pre-exocytotic events and explore the nature of somatic vesicle storage, transport, and docking at the membrane of serotonergic neurons from raphe nuclei of the rat brain. We find that the vesicles (or unresolved vesicular clusters) are quiescent (mean square displacement, MSD ∼0.04 μm^2^/s) before depolarization, and they move minimally (<1 μm) from their locations over a time-scale of minutes. However, within minutes of depolarization, the vesicles become more dynamic (MSD ∼0.3 μm^2^/s), and display larger range (several μm) motions, though without any clear directionality. Docking and subsequent exocytosis at the membrane happen at a timescale (∼25 ms) that is slower than most synaptic exocytosis processes, but faster than almost all somatic exocytosis processes observed in endocrine cells. We conclude that, (A) depolarization causes de-tethering of the neurotransmitter vesicles from their storage locations, and this constitutes a critical event in somatic exocytosis; (B) their subsequent transport kinetics can be described by a process of constrained diffusion, and (C) the pre-exocytosis kinetics at the membrane is faster than most other somatic exocytosis processes reported so far.

## Introduction

Monoaminergic neurotransmission is important for processes related to mood, memory, reward, and for neurodegenerative diseases such as Parkinson’s and Alzheimer’s (Carlsson, 1987; Nazarali and Reynolds, 1992; Kurian et al., 2011). Most monoaminergic neurotransmission is thought to occur through the synaptic exocytosis route. However, over the years, it has been shown that a substantial part of monoamine release may be extra synaptic (Descarries et al., 1975; Agnati et al., 1986; Bunin and Wightman, 1998; De-Miguel and Trueta, 2005; Fuxe et al., 2007; Kaushalya et al., 2008a; De-Miguel and Fuxe, 2012; Trueta and De-Miguel, 2012). Monoamine neurotransmitters in CNS neurons are contained in vesicles that are located not only at the synaptic terminals and in axonal processes, but also in the cell body (Coggeshall, 1972). Somatic release of neurotransmitters has been observed for more than three decades (Johnson and Pilar, 1980; Suetake et al., 1981; Agnati et al., 2010) and its quantal nature (suggesting vesicular release akin to synaptic exocytosis) was convincingly demonstrated in dopaminergic neurons of *Planorbis corneus* more than a decade ago (Chen et al., 1995). Subsequently, the phenomenon has been observed in multiple systems, including the dopaminergic neurons of rat and mice (Jaffe et al., 1998; Puopolo et al., 2001). This has also been observed in serotonergic neurons, such as the Retzius neurons of the leech (Bruns et al., 2000; Trueta et al., 2003, 2004; De-Miguel and Trueta, 2005), raphe neurons of the rat (Kaushalya and Maiti, 2008; Kaushalya et al., 2008a,c; Colgan et al., 2009), the mesencephalic trigeminal nucleus of the rat (Zhang et al., 2012), and in differentiated human embryonic stem cells (Kumar et al., 2009). De-Miguel and coworkers have identified the nature of the calcium channels which couple to the somatic exocytosis of serotonin (Trueta et al., 2003, 2004; De-Miguel and Trueta, 2005). However, not much is understood about the mechanism underlying this novel mode of exocytosis, and the role it may be playing in neurotransmission and neuromodulation. In addition to its yet to be discovered role in normal neurotransmission, somatic exocytosis potentially offers a powerful handle to therapeutically control mood disorders, substance addiction, and neurodegenerative diseases in the human brain. Moreover, the sheer size of the soma compared to that of a typical presynaptic terminal offers a unique advantage for understanding this mode of neurotransmitter exocytosis, especially the pre-exocytosis events. However, this aspect is yet to be exploited fully.

One of the recurrent questions is how the characteristics of somatic exocytosis compare to the more common processes of synaptic exocytosis. Synaptic exocytosis is characterized by several defining aspects. For example, synapses contain different pools of readily releasable and reserve set of vesicles (Murthy and Stevens, 1999). These vesicles are relatively uniform in size (∼40 nm diameter) and neurotransmitter concentration (which is typically about a few 100 mM). The process is critically controlled by the increase of intracellular Ca^++^ levels which occurs rapidly after depolarization (Katz and Miledi, 1970; Llinas et al., 1981). Exocytosis occurs at special regions of the synapse which are known as “active zones.” A number of proteins (such as CaM kinase II and synapsin I; Llinas et al., 1991) and networks of macromolecules, called “active zone materials,” are involved in the release from the cytoskeleton, and subsequent transport and docking of the vesicles during exocytosis (Szule et al., 2012). The residence time of the vesicles at the membrane can be as low as 10 ms (He et al., 2006), or even a fraction of a ms (Wolfel and Schneggenburger, 2003). The whole cycle of vesicle filling, priming, exocytosis, and endocytosis typically takes less than 30 s (Ryan et al., 1993).

Some of these aspects have been studied for somatic exocytosis and clear similarities and differences with synaptic exocytosis have been found. Somatic vesicles, as observed in transmission electron microscopy (TEM) images, are usually larger than their synaptic counterparts, and range from ∼80 to a few 100 nm (Coggeshall, 1972; Bruns and Jahn, 1995; Chen et al., 1995; Bruns et al., 2000; Puopolo et al., 2001; Trueta et al., 2003). They appear to be of two types, large dense core vesicles, and smaller clear vesicles, the later being similar to those found at synapses (De-Miguel and Trueta, 2005). Some of the somatic (clear) vesicles can be organized in multi-vesicular bodies, which seem to get processed into dense core vesicles near the Golgi (Trueta et al., 2012). The process of exocytosis is known to be Ca^++^ dependent (Johnson and Pilar, 1980; Puopolo et al., 2001; Trueta et al., 2003, 2004; Patel et al., 2009). Also the neurotransmitter concentration of the vesicles is fairly similar, though dense core vesicles contain about an order of magnitude larger number of molecules compared to the synaptic vesicles (Bruns et al., 2000). It has been shown that three photon microscopy can directly visualize serotonin (Maiti et al., 1997). In our laboratory, we have employed this technique to uncover further aspects of somatic exocytosis in mammalian serotonergic neurons. We have shown that somatic vesicles can be imaged in live neurons and their contents can be estimated (Balaji et al., 2005). This is facilitated by changes in the serotonin emission spectrum under vesicular conditions (Kaushalya et al., 2008b; Nag et al., 2008). Quantitatively, somatic exocytosis forms a substantial fraction of the total serotonin release in a rat brain section under depolarization (Kaushalya et al., 2008c). Three photon microscopy of serotonergic neurons of the raphe nucleus of the rat brain has revealed serotonin fluorescent spots with an average diameter of ∼370 nm with an estimated serotonin concentration of ∼100’s of mM (Kaushalya et al., 2008a; Nag et al., 2008). These individual spots could be large vesicles, or they can be unresolved clusters of multiple vesicles (Trueta et al., 2012). These serotonin fluorescent spots are distributed throughout the soma, but most seem to be arranged in a perinuclear fashion. These contents get exocytosed without any specific dependence on their storage locations, and they show no clear evidence of the presence of a reserve pool of vesicles. In low time resolution measurements (one frame per several minutes) the serotonin fluorescent spots appear fairly stationary at their “storage” locations until they stochastically disappear upon serotonin exocytosis. Later work using multiphoton microscopy has suggested that newly recycled serotonin vesicles release faster than a pre-existing reserve pool (Colgan et al., 2009). Also, a Total Internal Reflection Fluorescence (TIRF) study shows that exocytosis happens fairly uniformly throughout the basal cell membrane (through which the cell adheres to the cover glass), without any apparent active zone (Kaushalya et al., 2008a). However, the dynamics of vesicle release from the storage location, transport to the site of exocytosis, and docking events at the membrane have not been explored so far.

Here we employed faster imaging, using three photon and TIRF microscopy, to better resolve the events preceding exocytosis. We examined the vesicular dynamics associated with the process of exocytosis induced by depolarization with high K^+^. A unique aspect of somatic exocytosis that distinguishes it from its synaptic counterpart is that somatic vesicles have to traverse a relatively long path (many μm) from the inside of the cell to the plasma membrane before exocytosis. This transport can in principle be diffusive or active. We analyzed individual tracks of serotonin fluorescent spots as they proceeded toward exocytosis to address this issue. We also measured the average residence time at the membrane with TIRF microscopy of FM 1-43 labeled vesicles. This gives an idea about the time it takes to assemble the exocytosis machinery at the site of exocytosis on the cell membrane and the time required for the pore to open. These are expected to be parameters which distinguish between synaptic exocytosis and somatic exocytosis observed in endocrine cells.

## Materials and Methods

### Primary culture of raphe neurons

The details of this procedure have been given earlier (Kaushalya et al., 2008a). All animal handling procedures were approved by the Animal Ethics Committee of the institute. In brief, female neonatal (P0–P2) Wistar rats were used for the cultures. The rostral brain stem containing a major portion of the raphe nuclei was dissected and put in ice-cold Thomson’s buffer (146 mM NaCl, 5.4 mM KCl, 1.8 mM CaCl_2_, 0.8 mM MgSO_4_, 0.4 mM KH_2_PO_4_, 0.3 mM Na_2_HPO_4_, 5 mM dextrose, and 20 mM Na-HEPES, pH adjusted to 7.35).This was followed by Trypsin (0.025%) digestion for 30 min at 37°C, trituration, and plating on poly l-ornithine coated coverslip-bottomed Petri dishes. Cells were grown in glial conditioned medium [60% DMEM (containing 10% FBS) + 40% Neurobasal-A with B27 supplement and penicillin/streptomycin]. The medium was conditioned for 1 day with glial cultures. Basic fibroblast growth factor (b-FGF; 0.5 ng/ml) and fibroblast growth factor 5 (FGF-5; 1 ng/ml) were also added to enhance the survival of cells. Medium was changed every 48 h. Images of the cells were recorded after 5 days using Thomson’s solution as the buffer. The protocol led to serotonergic cultures mixed with other types of neurons and glia. A large majority of the neurons were of serotonergic type, which was easy to identify by serotonin auto-fluorescence (∼350 nm). The cell culture components were purchased from Sigma (FGF and FGF-5), Gibco (Neurobasal-A, B27), and Himedia (India; DMEM and FBS). Buffer salts were purchased from S. D. Fine-Chem, Ltd. (India).

### Brain slices preparation

Female neonatal Wistar rats (P2–P3) were used for obtaining brain slices. One hundred μm slices from the dorsal raphe region were cut with a microtome (Model 3000, Vibratome, St. Louis, USA). All dissections were performed in ice-cold oxygen bubbled Thomson’s buffer. The brain slices were transferred to cover slip-bottomed Petri dishes and imaged in Thomson’s solution.

### KCl induced depolarization of neurons

Depolarization was induced by a modified Thomson’s buffer (pH 7.35), where a part of the Na^+^ ions was replaced with K^+^ ions, and extra Ca^++^ was added, yielding a concentration of 100 mM K^+^ and 16 mM Ca^++^ in the depolarizing solution. 500 μl of this solution was added to 500 μl of existing Thomson’s buffer in the Petri dish, resulting in a final concentration of 50 mM K^+^ and 8 mM Ca^++^.

### FM 1-43 staining of vesicles

Vesicle staining of cultured neurons was performed following published protocols for FM 1-43 dye staining, with some modifications (Betz et al., 1992; Brumback et al., 2004). The cultured primary serotonergic neurons were depolarized using depolarizing solution containing 5 μM FM 1-43. The exocytosis buffer was removed and replaced by normal Thomson’s buffer after 10 min and the cells were left for 20 min in this buffer. This allowed the cells to repolarize and internalize the FM 1-43-loaded vesicles. FM 1-43 staining of the outer membrane of the cell was removed by washing three times with normal buffer. The cells were then depolarized again, resulting in exocytosis of vesicles loaded with FM 1-43, which were imaged using confocal or TIRF microscopy.

### Multiphoton imaging of serotonin

The multiphoton microscope set-up based on a modified MRC600 confocal microscope (BioRad, UK) has been described elsewhere (Balaji et al., 2005). Briefly, a Ti:Sapphire (MIRA, Coherent, Inc., USA) laser operating at 740 nm and producing ∼100 fs pulses (repetition rate, 76 MHz) caused three photon excitation of serotonin. The radiation was separated from the serotonin fluorescence by a dichroic mirror (670dcxruv, Chroma, USA), which was subsequently passed through a 1-cm thick liquid CuSO_4_ emission filter. The filtered signal was detected externally by an analog photomultiplier tube (Model: P30A-01 Electron Tubes Limited, UK). For recording the movements of the vesicles, we performed three photon imaging of a single optical slice (taken at about the middle of a cell) continuously at the rate of 3 s/frame. This experiment is performed in primary cultures and also in brain tissue slices.

### Analysis of time series movies

The analysis of the time series obtained by three photon microscopy of serotonergic neurons was performed using the Object Tracker wizard of the Huygen’s Professional software (Scientific Volume Imaging, B.V Netherlands). By manually selecting the serotonin fluorescent spots and background regions, the Object Tracker was trained to differentiate between the objects and the background and to track the motion of the spots. Thresholding was performed on the brightness of the objects. Analysis was performed for the spots for which movement of the calculated center of mass of the objects (spots) were 2 μm or less per time frame. The data were analyzed, frame by frame, for the position and displacement, the *x* and *y* velocity of the tracked objects and the mean squared displacement (MSD) of the tracked objects. MSD was calculated by measuring the square of the distance traveled by a given spot from the origin and then dividing it by the time elapsed. Subsequently, this quantity is averaged over all the spots. The Huygens program does this automatically. Analysis was performed on a total of 149 fluorescent spots before and 137 spots after depolarization from six cells. We note that the upper bound for the movement is somewhat arbitrary, and it can allow two different spots to be counted as one in some cases. However, this should happen equally both before and after depolarization, unless the total movement before depolarization is small compared to that after depolarization. Therefore, any inference with respect to changes in vesicle dynamics should be robust with respect to such errors.

### Quasi-simultaneous imaging of serotonin and FM 1-43

Femtosecond pulsed 740 nm light was used to excite serotonin (as described) and a continuous wave (CW) beam of 543 nm (from a He-Ne laser) was used for FM 1-43. Both were aligned along the same paths. The same cell (of primary cultured neurons) was imaged quasi-simultaneously by changing the dichroics for serotonin (detected using a external non-descanned detector) and FM 1-43 (detected using the internal detector, passed through a 605/75 emission filter), and switching the lasers. A 1-cm thick solution of Tetramine copper sulfate solution was used in front of serotonin detector to filter out any fluorescence coming from FM 1-43. For both serotonin and FM 1-43, *Z*-stacks with 1 μM separation were recorded before and after depolarization. The images were analyzed using the ImageJ software (open source, available from the website: http://rsbweb.nih.gov./ij/). Corresponding single *Z*-plane images for serotonin and FM 1-43 were analyzed for co-localization and reduction of brightness following depolarization.

### Total internal reflection microscopy

The details of the objective-based TIRF microscope set-up have been described elsewhere (Kaushalya et al., 2008a). Briefly, a 532-nm laser excitation beam (Verdi V-10; Coherent, Inc.) was focused at the back focal plane (BFP) of the objective (Olympus Apo 3100 1.65 NA, oil objective) with a 30-cm focal length lens in an inverted microscope (Olympus IX-71). The beam was moved along the diameter of the BFP of the objective so that the beam emerged from the objective at high angles, undergoing total internal reflection at the coverslip – water interface. The emission was separated from the excitation by a 535 nm long-pass dichroic (Chroma, USA) and filtered by a 605/75 nm emission filter (Chroma, USA). Images were captured using an Electron Gain Multiplier CCD (EM-CCD) camera (Andor, Ixon; model No. DV887ECS-UVB, Ireland). Special objective oil (refractive index: η_oil_ = 1.78; Cargille, USA) was used for imaging, and the cells (primary serotonergic neurons) were cultured on special coverslips (η_coverslip_ = 1.65; Olympus, Singapore). The penetration depth was ∼100 nm (estimated by the gradual withdrawal of a 20 nm fluorescent bead).

## Results

### Dynamics of vesicles before and after depolarization

**Figure 1:**
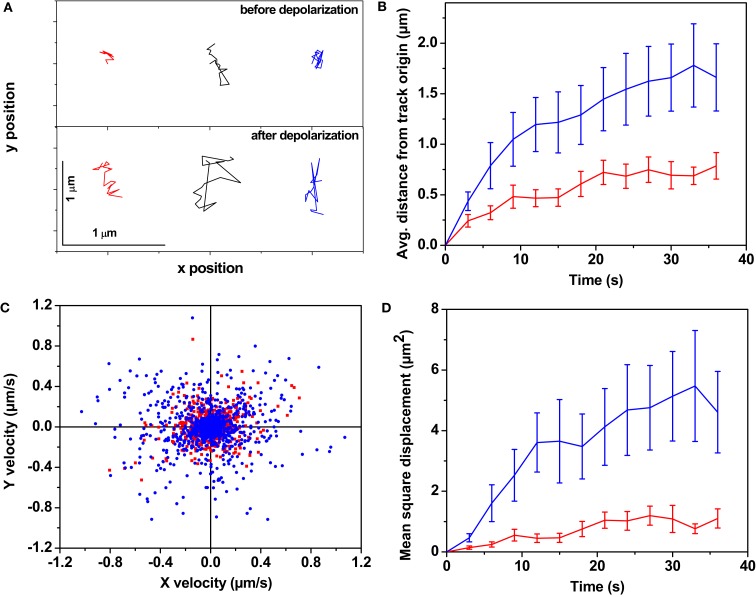
**The dynamics of somatic vesicles pre- and post-depolarization**. Three photon excitation microscopy shows dynamics of somatic serotonergic vesicles (or unresolved vesicular clusters) in the cell body and in the processes before and after K^+^-induced depolarization. **(A)** Individual tracks of a few serotonin fluorescent spots before (top) and after (bottom) depolarization. In the following, red and blue denote measurements performed before and after depolarization respectively. **(B)** Average displacement from the track origin vs. time, **(C)** Velocity distribution obtained from the tracks, **(D)** Mean squared displacement vs. time. (Error bars correspond to standard errors of the mean. *N* = 149 fluorescent spots before depolarization and *N* = 137 spots after depolarization from six cells).

Figure 1 represents the analysis of the dynamics of the serotonin fluorescent spots obtained from a total of six serotonergic neurons recorded in three different experiments (once with primary cultured neurons and twice with raphe brain slices). A time series of a single focal plane was recorded from the neurons before depolarization (images were recorded every 3 s). Another time series was recorded from the same plane under depolarizing conditions starting 10 min after excess KCl addition. Individual spots were tracked as described in the Section “Materials and Methods.” Figure 1A shows tracks obtained from a few representative spots before (Figure 1A top panel) and after (Figure 1A, bottom panel) depolarization with high K^+^. The tracks in the upper panel show that the serotonin fluorescent spots perform very limited movement about their starting points. The tracks from the same fluorescent spots (in corresponding colors in the bottom panel) show that they move over a much larger range after depolarization. This is obvious also from the plot of average distance from the track origin of all identified tracks as plotted in Figure 1B. Before depolarization, the spots moved less than a μm (red) from their origin in ∼30 s (∼5 min in some cases), while after depolarization (blue) they moved considerably more within the same time. We also asked whether this movement is passive or active, and random or directed. Figure 1C analyzes the directionality of the motion by plotting *y* velocity vs. *x* velocity, with the velocities computed at each step. The plot displays a nearly circular symmetry, which implies that there is no correlation between the *x* and the *y* velocities, indicating random movement. Finally, the MSD was calculated from the tracks (Figure 1D). The MSD was quasi-linear in the beginning, indicating a random diffusive motion (Berg, 1993) after depolarization. However, in about 15 s, the MSD values start to show a plateau. Such a plot indicates some degree of confinement (Daumas et al., 2003) within a spatial scale of several microns. This implies that at any instant, the particles are free to randomly move about within a short spatial range, but cannot go as far as a freely diffusing particle would reach at longer timescales. The MSD value per unit time calculated from the initial slope (first 12 s) was ∼0.04 μm^2^/s before KCl addition (Figure 1D, red), while it increased to ∼0.3 μm^2^/s after the addition (Figure 1D, blue).

**Figure A1:**
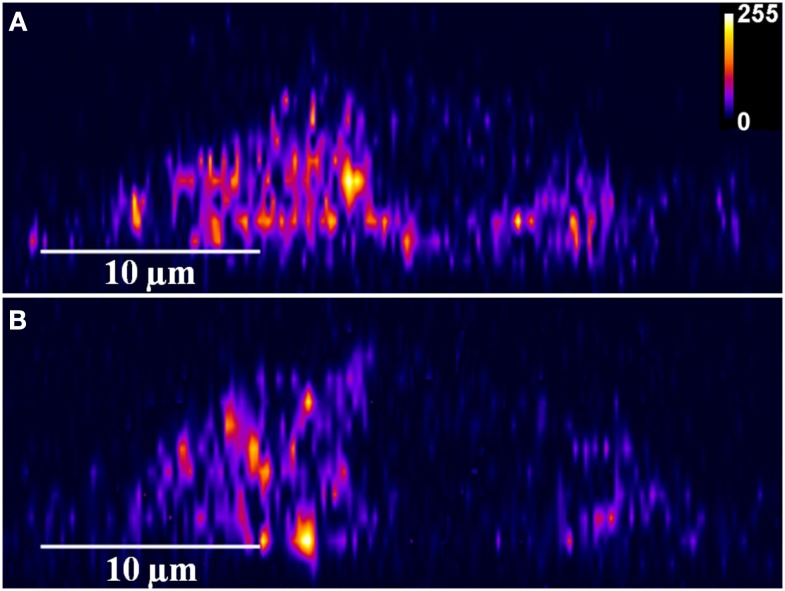
**Depolarization induces serotonin exocytosis**. *X*-*Z* slices of three photon serotonin images of a single cell (of cultured serotonergic neurons) **(A)** before and **(B)** 20 min after depolarization (Intensities are false color coded).

The dynamics before and after the stimulation can be better observed through the Videos S1 and S2 in Supplementary Material, which are recorded from brain tissue before and 10 min after depolarization respectively. In Video S2 in Supplementary Material, the numbers of fluorescent spots are smaller, likely due to exocytosis in the intervening time. Of course, the disappearance of serotonin fluorescent spots from a single optical slice may also be due to the movement of the spots along the *z* (depth) direction. However, volume imaging of the cells before and after depolarization shows that the spots have indeed disappeared after depolarization (Figure A1 in Appendix). We note that some of these data recorded at lower time resolution has been published elsewhere (Kaushalya et al., 2008a). Earlier work involving imaging of serotonergic cells in RN46A cell line as well as primary culture and Raphe tissue of rat brain at lower time resolution showed that the somatic exocytosis of serotonin upon KCl induced depolarization is rather slow and happens over a timescale of tens of minutes (Balaji et al., 2005; Kaushalya et al., 2008a).

### Co-localization of serotonin and FM 1-43

**Figure 2:**
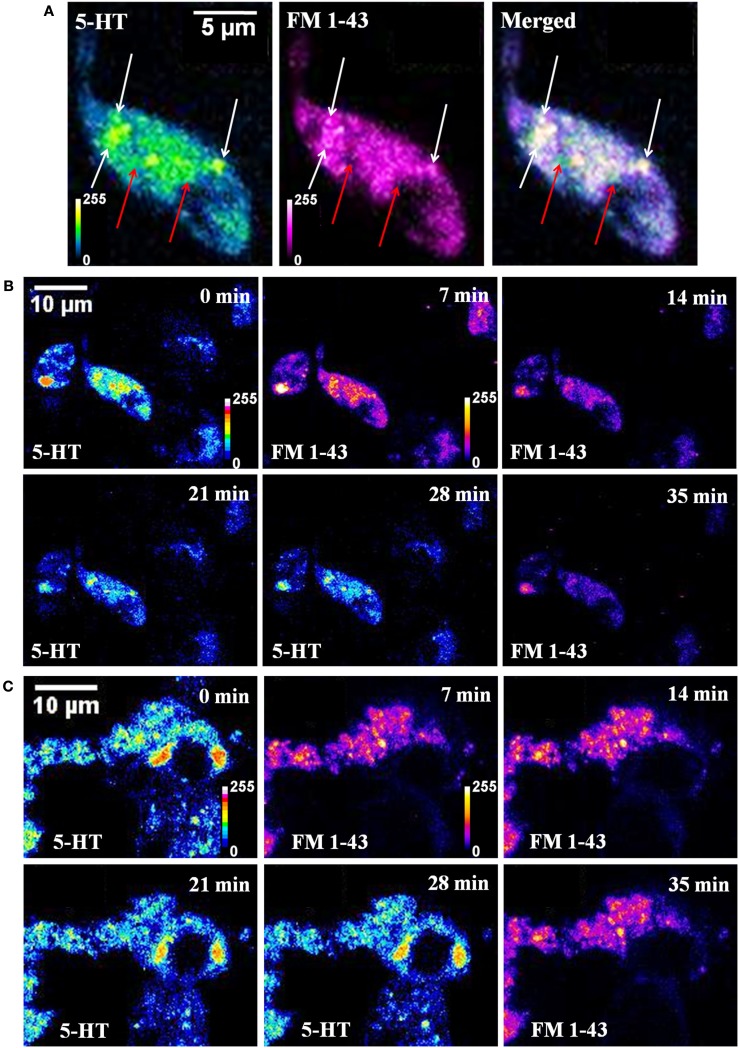
**Co-localization of serotonin and FM 1-43 labeled vesicles**. **(A)** Serotonin (5-HT, blue green) and FM 1-43 (FM 1-43, magenta) images of a cultured serotonergic neuron. In the merged image, white arrows show co-localized vesicular structures, and red arrows mark spots visible in the serotonin channel only. **(B,C)** Time lapsed quasi-simultaneous imaging of serotonin (5-HT) and FM 1-43 in depolarized **(B)** and sham-treated control cells. **(C)** Top right corner of each image shows the starting time of image recording following treatment. All image intensities are false color coded.

**Figure A2:**
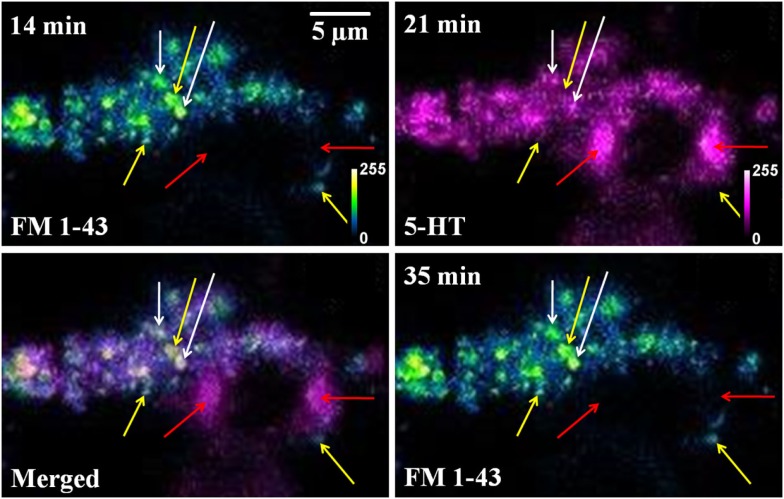
**FM 1-43 and serotonin co-localization is not caused by cross-talk between fluorescence channels**. FM 1-43 (blue green) and Serotonin (5-HT, magenta) images of the same cultured serotonergic neuron. In the merged image (FM 1-43, 14min and 5-HT, 21 min) white arrows show co-localized vesicular structures (recycled vesicles), yellow arrows mark spots visible only in the FM 1-43 channel, and red arrows mark spots visible only in the serotonin channel. FM 1-43 images recorded before (14 min) and after (35 min) serotonin imaging shows the same bright spots (by yellow arrows), but these are absent in the serotonin channel (5-HT, 21 min). Top left corner of each image shows the starting time of image recording following sham treatment (Intensities false are color coded).

**Figure A3:**
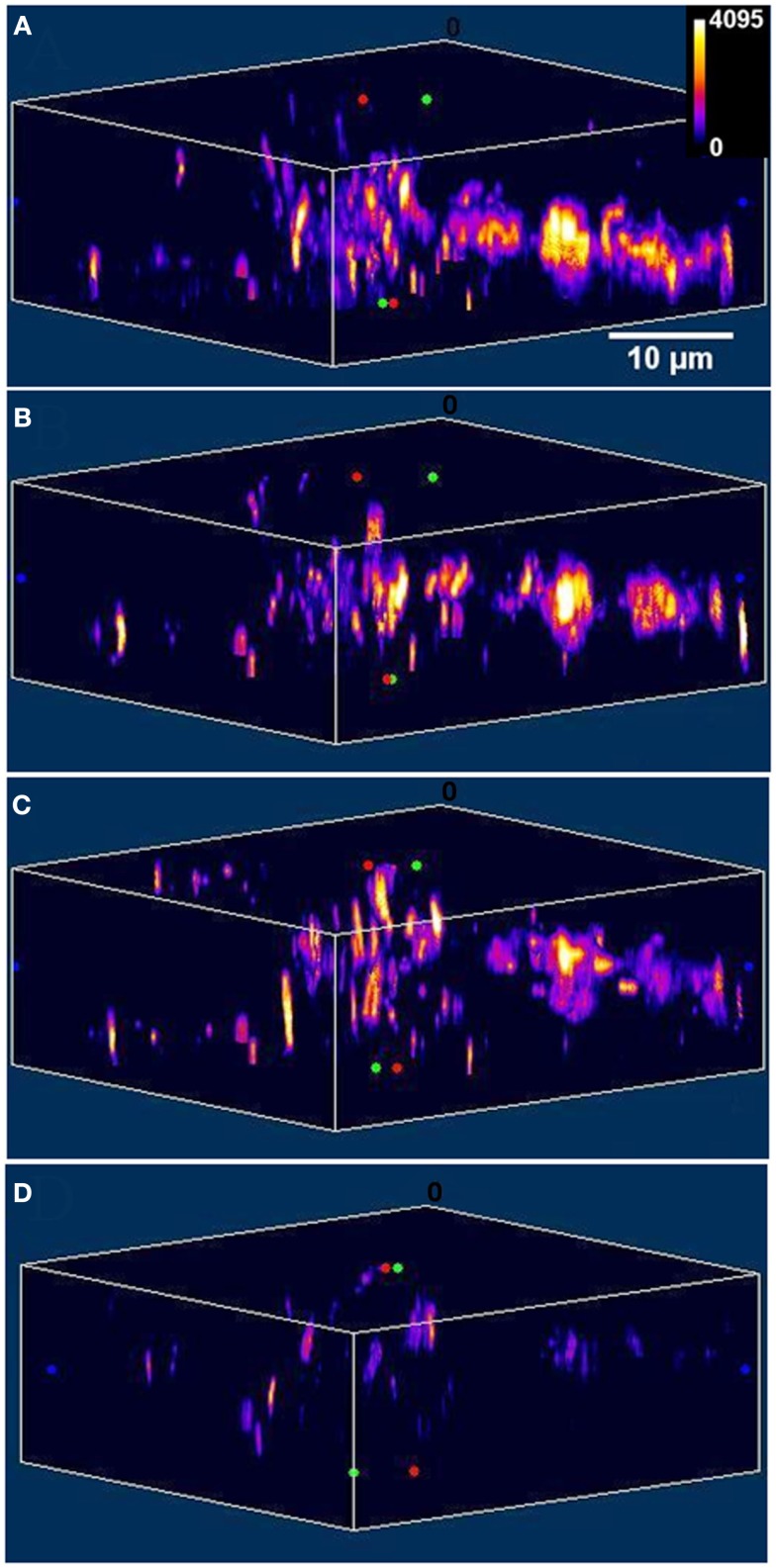
**Depolarization induces exocytosis of FM 1-43 labeled vesicles**. *X*-*Z* slices of the images of FM 1-43 labeled vesicles in a cultured serotonergic neuron **(A)** before, and **(B–D)** 5, 15, and 30 min after depolarization respectively (Intensities are false color coded).

**Figure A4:**
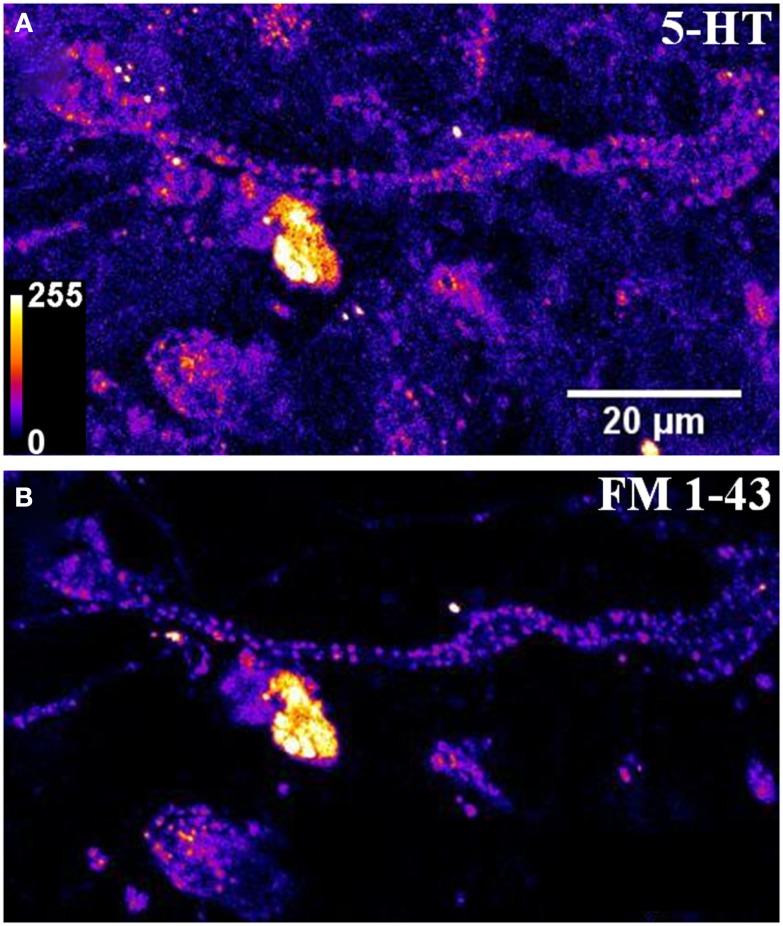
**A large area image of serotonergic cells shows a high degree of co-localization between (A) three photon image of serotonergic vesicular clusters and (B) confocal image of FM1-43 labeled spots**. Most spots observed in the FM1-43 channel co-localize with bright spots observed in the serotonin channel, though there are entire cells in the serotonergic channel which do not appear to take up the dye FM 1-43 (Intensities false are color coded).

A co-localization study was performed to check whether the endosome labeling dye FM 1-43 used for the TIRF measurements did actually label the serotonin spots. Three photon imaging of serotonin and conventional confocal imaging of FM 1-43 was performed on the same cell (of cultured serotonergic neurons) quasi-simultaneously. Figure 2A shows a single *Z*-plane image of a cell. FM 1-43 labeled vesicles (or unresolved vesicular clusters, magenta) and serotonin fluorescent spots (5-HT, blue green) show good co-localization in the merged image (some of them are marked by white arrows). A few serotonin spots (marked by red arrows) do not co-localize with FM 1-43. These most likely mark the vesicles that did not undergo recycling. This can be better observed in the Figure A2 in Appendix from a non-KCl treated cell where the red arrows show bright serotonin spots that are not observed in the FM 1-43 channel. Yellow arrows (Figure A2 in Appendix) show spots bright in the FM 1-43 channel but not in the serotonin channel, ruling out the possibility of FM 1-43 leakage into the serotonin channel. There is a finite possibility that a spot in one channel is not seen in the other due to the movement of the spots within the intervening time. This possibility can be ruled out by imaging one channel both before and after the other channel. Figure A2 in Appendix demonstrates this issue. Yellow arrows mark the same bright spots that were observed in the FM 1-43 channel (not co-localized with serotonin) recorded before (panel 1, 14 min) and after (panel 4, 35 min) serotonin imaging (panel 2, 21 min). These are possibly vesicles that have not been refilled with serotonin. Conversely, red arrows mark spots that are bright in the serotonin channel (panel 2, 21 min) but are absent in the FM 1-43 channel both before (panel 1, 14 min) and after (panel 4, 35 min) serotonin imaging. These are possibly non-recycled vesicles. The white arrows on the other hand show the spots with good co-localization. Figure 2B shows reduction of brightness in both serotonin (5-HT, false-colored according to green-fire-blue look up table or LUT) and FM 1-43 (in fire LUT) channels after high K^+^ induced depolarization, while in the absence of depolarization the brightness does not reduce significantly over the same timescale (Figure 2C). This indicates that FM 1-43 indeed labeled the serotonin fluorescent spots, and the recycled vesicles can undergo exocytosis. Analysis of serotonin brightness shows a reduction of ∼36% over 20 min (Figure 2B, 5-HT 0 vs. 21 min) and ∼46% in 30 min (Figure 2B, 5-HT 0 vs. 28 min) following depolarization. These values are similar to those reported earlier by us (Balaji et al., 2005; Kaushalya et al., 2008a). Comparatively in the sham-treated control cells it is ∼10% in 30 min (Figure 2C, 5-HT 0 vs. 28 min). On the other hand, FM 1-43 brightness decreases by ∼47% from 7 to 14 min following depolarization and by ∼73% from 7 to 35 min (Figure 2B). This larger depletion is at least partly due to the higher level of background signal observed in the serotonin three photon images. However, this probably also indicates that the newly recycled vesicles are more readily exocytosed, which has been reported earlier (Colgan et al., 2009). For the sham-treated control cells (Figure 2C, FM 1-43 7, 14, and 35 min) the change in FM 1-43 brightness is negligible, indicating photobleaching is not a major issue. *X*-*Z* slice of a single cell imaged for serotonin before (Figure A1A in Appendix) and 20 min after depolarization (Figure A1B in Appendix) shows that the loss of fluorescent spots has no preferred location. This observation is also true for the recycled vesicles, as can be seen in a similar *X*-*Z* slice of another cell imaged with FM 1-43 (Figures A3A–D in Appendix). These results indicate that a considerable fraction of the spots observed in the FM1-43 channel likely represents serotonergic vesicles, although some other organelles may also get labeled. A separate larger area image is presented in Figure A4 in Appendix to further ascertain the degree of co-localization. A large number of spots are observed in the serotonin channel and also in the FM1-43 channel. Vast majority of the spots observed in the FM1-43 channel appear to be nearly perfectly co-localized with those observed in the serotonin channel (though some cells observed in the serotonin channel hardly take up the FM 1-43 dye), suggesting that at least a large fraction of the vesicles/vesicular clusters stained by FM1-43 indeed contains serotonin.

### TIRF microscopy of vesicle dynamics at the membrane

**Figure 3:**
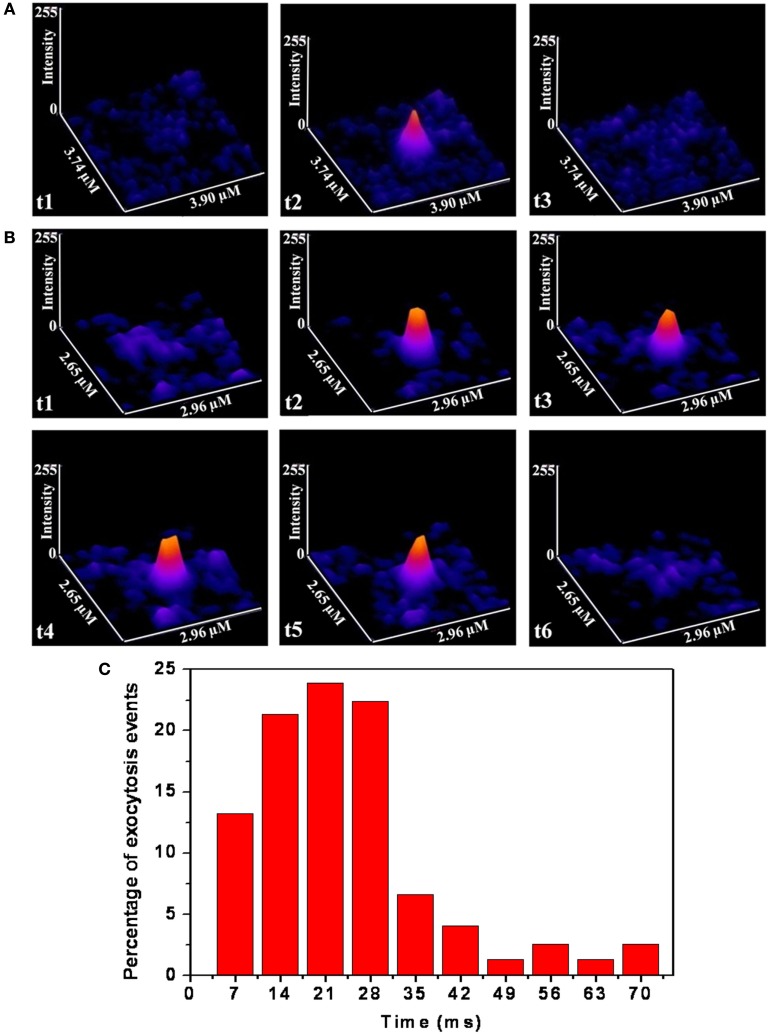
**TIRF images of somatic vesicles showing time spent at the membrane**. **(A,B)** Montage of consecutive frames (each frame is 7 ms) showing individual release events following depolarization which last for 1 **(A)** and 4 **(B)** frames respectively. (Image intensities are false color coded) **(C)** Histogram of the percentage of events vs. docking times (*N* = 75 spots from three cultured serotonergic neurons).

FM-1-43 labeled vesicles (or vesicular clusters) of cultured serotonergic neurons were imaged with a home-built TIRF microscope. TIRF imaging was performed under depolarizing condition at a fast rate (down to 7 ms/frame) to resolve the time that the vesicles spend “docked” at the membrane on an average. The depth resolution of the microscope was about 100 nm, so that “docking” in this context merely refers to their being present within this distance from the membrane. Actual time spent at the membrane would be less than this. We observed that while some of the FM 1-43 labeled vesicles are present only in a single frame, most are present in more than one frame. For example Figure 3A shows a vesicle which is visible for one frame only (see frame t2; t1 and t3 recorded at the same place do not show the spot, where t1, t2, t3 are three consecutive frames separated by 7 ms each). Figure 3B shows another vesicle which is visible over four frames or about 28 ms (see t1–t6). We note that the vesicles were almost always observed to disappear completely at a single step (e.g., Figure 3A, t3; Figure 3B, t6), without any intermediate step where the vesicle appears dim. Since the vesicles on an average move about 100 nm/s (Figure 1B), it is unlikely that they will move out of the TIRF range of 100 nm within one frame (7 ms). Therefore the single step disappearance suggests that we are actually observing exocytosis of vesicles which are docked at the membrane. A histogram of the residence times at the membrane is plotted in Figure 3C. It shows a peak at 21 ms, and the average is about 25 ms (obtained from the analysis of 75 spots from three cells). Since the peak of the distribution is well resolved in the histogram, we infer that the time resolution is adequate for a reliable estimate of the residence time. We note that in this analysis, any spot observed with reasonable intensity in a frame is assumed to have resided for the full length of time in that frame.

**Figure A5:**
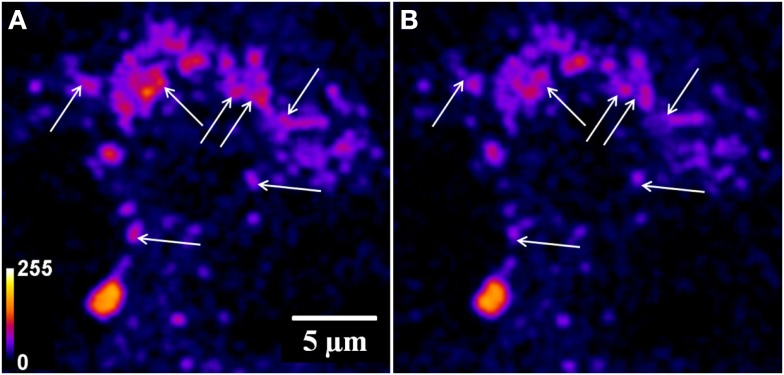
**Evidence for individual vesicles leaving a cluster during exocytosis**. A three photon serotonin image of a cell at high zoom recorded **(A)** before and **(B)** 21 min after depolarization. The arrows mark vesicular clusters which appear to lose a fraction of the constituents during exocytosis (Intensities false are color coded).

We note that what we observe in the serotonin channel are most likely vesicular clusters, but what we observe with TIRF is disappearance of individual vesicles. It is possible that during exocytosis, individual vesicles independently leave their host clusters. This should result in a step-wise reduction of the cluster brightness, and for clusters which are larger than the resolution limit, also their size. Indeed, for many of the cases we do observe that vesicle clusters lose their brightness post-depolarization, and at least in some cases it is evident that the clusters shrink in size in a step-wise fashion. This is highlighted in the Figure A5 in Appendix which presents a zoomed up three photon image of an exocytosing cell (see vesicle clusters marked with arrows, before and after depolarization).

## Discussion

### Dynamics of vesicles before and after depolarization

The ability to visualize neurotransmitter vesicles/vesicular clusters with their auto-fluorescence provides an unprecedented opportunity to track them in real time, anywhere in the cell and at any time, until their exocytosis. The technique of labeling vesicles by FM dyes, following the pioneering work by Betz et al. (1992), has made it possible to follow the dynamics of freshly recycled vesicles. However, this technique does not allow one to follow the vesicle content, or the movement of the non-recycled, stored original vesicles. Our observations using the three photon microscopy technique allows us to specifically look at all the neurotransmitter vesicles inside the cell. We observe that the predominant fraction of the somatic serotonin fluorescent spots are quiescent and appear to be attached to cellular structures which prevent substantial movement. They can remain so for rather long times (longer than 5 min). However, depolarization induces their release from captivity within minutes. These spots become much more dynamic, and they may move over several microns with randomly directed motion. We next ask if this random motion can be explained by passive diffusion. The hallmark of passive diffusion is a randomly directed movement with a mean square displacement that is proportional to time. The initial part of the mean square deviation plots are indeed reasonably linear (Figure 1D). However, it later tapers off to a plateau, indicating a process of constrained diffusion (Daumas et al., 2003). This implies that the vesicular motion is bounded by local cellular structures, as can be expected from a relatively large object of this size. We note that similar observations of undirected vesicle motion have been made with GFP-tagged neurotransmitters in drosophila neuromuscular junctions (Shakiryanova et al., 2005). It is pertinent to ask whether the observed numerical values are consistent with passive diffusion. The diameter of the serotonin fluorescent spots is about 370 nm (Kaushalya et al., 2008a), and their shape is expected to be spherical. In aqueous solutions this would translate to a diffusion constant (*D*) of 1.2 μm^2^/s. If the cytosolic viscosity is about 3 times that of water (Swaminathan et al., 1997) then *D* would be about 0.4 μm^2^/s. The calculated *D* in the cytosol from the initial slope after depolarization is 0.3 μm^2^/s, which is similar. This value is somewhat larger than that reported for neurotransmitter vesicles in retina cells (0.11 μm^2^/s; Rea et al., 2004), and much larger than that observed in frog neuromuscular junctions (Gaffield et al., 2006). However, the relative increase of mobility after excitation (about 7×) is similar to that reported by them (Gaffield et al., 2006). This suggests that a passive diffusion can explain the observed movements, though there are strong boundary effects that limit diffusion to spatial scales of a few μm. We note that our data do not exclude the possibility that the vesicles/clusters are tethered to the cytoskeleton in a way that allows them a range of relatively fast random movements, while they proceed slowly toward the plasma membrane (Trueta et al., 2012).

### Docking times

Exocytosis requires docking, priming, and pore opening at the membrane, and also requires the assembly of a large number of vesicular and plasma membrane protein molecules. The residence time at the membrane therefore reflects the total time required for these assembly processes. This process can take very different times in different types of cells. A comparison table between different experiments is given in the (Table A1 in Appendix). Residence time at the membrane (referred to here as “docking time”) can be a useful parameter to characterize the nature of vesicular exocytosis. Studies with various techniques such as capacitance measurement, amperometry, Ca^++^ uncaging, and fluorescence imaging yield a range of synaptic vesicle exocytosis timescales. Neuronal exocytosis processes range from about 0.6 ms in rat CNS neuron synapse (Wolfel and Schneggenburger, 2003) to about hundreds of milliseconds in the Calyx of Held neuron (He et al., 2006). This timescale is typically faster than what can be followed with three photon microscopy. We therefore follow this process by labeling the vesicles with FM 1-43, and using fast video microscopy in the TIRF mode to analyze the dynamics. Our co-localization studies prove that a large fraction of the structures that we observe with three photon microscopy are indeed labeled by FM 1-43. We have also shown earlier (from slower time-scale data) that the rate at which the FM 1-43 labeled vesicles appear goes up by an order of magnitude after KCl administration, clearly indicating that what we observe is a depolarization-driven exocytosis process (Kaushalya et al., 2008a). Our observation of docking timescale of 25 ms is amongst the slower category of exocytosis processes observed in the synapses. On the other hand, this timescale is much faster than those typically observed in endocrine cells (such as chromaffin cells; Albillos et al., 1997). The timescale is faster than even the very fast somatic exocytosis observed in melanotrophs (Thomas et al., 1993) and gonadotropes (Zhu et al., 2002), and is similar to the vesicle fusion timescale observed in artificial lipid bilayers (Liu et al., 2005). Thus it is amongst the fastest, if not the fastest, somatic exocytosis process observed so far.

## Conclusion

We find that the kinetics of somatic exocytosis in serotonergic neurons has three main phases. Before depolarization, the vesicles/vesicular clusters appear confined. Post-depolarization, they are released from their storage locations and become much more dynamic, though their motion still appears to be constrained. The vesicles subsequently reach their final destination at the membrane, possibly as individual vesicles which leave the host clusters. No preferred location for exocytosis was observed on the membrane. The final process of docking and exocytotic release of neurotransmitters takes place in ∼25 ms, which is much more rapid than somatic exocytosis in endocrine cells and as rapid as many types of synaptic exocytosis.

## Conflict of Interest Statement

The authors declare that the research was conducted in the absence of any commercial or financial relationships that could be construed as a potential conflict of interest.

## Supplementary Material

The Supplementary Material for this article can be found online at http://www.frontiersin.org/Membrane_Physiology_and_Biophysics/10.3389/fphys.2012.00414/abstract

Supplementary Video S1 **Three photon excitation microscopy of a single optical slice shows false color intensity coded images of serotonin fluorescence spots in the cell body and in the processes of a serotonergic neuron in a cultured brain-slice containing the raphe, before depolarization**. Twenty frames recorded at 3 s/frame.

Supplementary Video S2 **Three photon excitation microscopy of the same plane 10 min post-depolarization**. Thirteen frames recorded at 3 s/frame.

## Appendix

**Table A1:** **Timescales of exocytosis**.

Cell type	Vesicle size (or type)	Release time	Technique
Melanotrophs (Thomas et al., 1993)	Dense core vesicle	40 ms	Capacitance
Leech motor neuron (Bruns and Jahn, 1995)	Small clear vesicle	260 μs	Amperometry
	Large dense core vesicle	1.3 ms	
Chromaffin (Albillos et al., 1997)	420 nm	190 ± 20 ms	Patch amperometry
Pancreatic β cells (Takahashi et al., 1997)	70–1000 nm	85–2000 ms	Capacitance measurement
PC 12 cells (Ninomiya et al., 1997)	Small vesicle	30 ms	EPSC measurement
Goldfish retinal bipolar neuron ribbon synapse (Zenisek et al., 2000)	30 nm	210 ± 30 ms	TIRF, FM 1-43
Inner hair cell (Beutner et al., 2001)	Small vesicle	1 ms	Capacitance
Gonadotrope (Zhu et al., 2002)	Dense core vesicle	30 ms	Capacitance
Rat CNS neuron synapse (Wolfel and Schneggenburger, 2003)	–	0.6 ms	Capacitance measurement, Ca^2+^ uncaging
Hippocampal pyramidal cell/vesicle recycling (Gandhi and Stevens, 2003)	–	Three modes: Fast: 400–860 ms	SyPHluorin, WF fluorescence
	Slow: 8–21 s		
Stranded: >45 s *In vitro* lipid bilayer (Liu et al., 2005)	50 nm	25 ms	TIRF, TMR
Calyx of held neuron synapse (He et al., 2006)	30–80 nm	10 ms – 1 s, 273 ± 22 ms	Capacitance measurement

*Comparison showing the size (or type) of the vesicles and the time of exocytosis in different types of cells*.
